# News and Notes

**Published:** 1899-12

**Authors:** 


### *NEWS AND NOTES.*

Dr. Geo. Smith died in Atlanta on November 4th.

Dr. Thomas F. Jones died near Cartersville, Ga., on November 13, of angina pectoris. Dr. Jones was one of the best known and most popular citizens in that community.

A bill is to be introduced at the present meeting of the Georgia Legislature compelling all Medical Colleges in the State to adopt a four-years graded course. We heartily indorse such an action by the Legislature.

The Charlotte Medical Journal is to be congratulated on becoming the "Official Organ" of the Medical Society of Virginia. We notice, however, that the Medical Register of Richmond, Va., also publishes some of the same articles.

An epidemic of typhoid fever has been reported as existing in the Kentucky University at Lexington. The cause was at first thought to be the drinking-water, but later it was thought to be milk from cows fed on distillery slop.

It is stated that the Maryland Medical Journal will be changed from a weekly to a monthly publication. Dr. John S. Fulton, formerly editor of the Bulletin of Johns Hopkins Hospital, will be the editor, in place of Dr. Canfield, resigned.

Prof. Lombroso has at last discovered a man of genius whom he can acquit of the charge of insanity. It is the memory of

Goethe which he has just honored in a recent article in an Italian literary journal. Goethe, he considers, was a normal, healthy and sublime poet.

---

Richmond, Va., is to have one of the largest and most elegantly equipped hospitals in the South. It is to cost between $150,000 and $200,000, and nearly all of the amount has already been subscribed. Dr. George Ben Johnston will be at the head of the Medical Department.

---

Here is what Moritz Ellinger, Esq., a distinguished medico-lawyer says of Christian Science: "Attention has been called to a new school of medical practice which, however, seems to be in closer alliance with the vocation of the undertaker than that of the ordinary pupil of Æsculapius."

---

The next meeting of the Southern Surgical and Gynecological Association will be held in New Orleans on December 5th, 6th and 7th, under the presidency of Dr. Joseph Taber Johnston of Washington, D. C. This is one of the best associations in the country and is well worthy to be called Southern.

---

Kussmaul of Vienna recently declined to sign the requirements provided for the examination to practice medicine, because the students were not taught the practical use of hydrotherapy and hygiene, which he regards as the most important, as well as the most neglected, department of medical teaching and practice.

---

Dr. John G. Clark, associate in gynecology at the Johns Hopkins University, has been elected professor of gynecology in the University of Pennsylvania, to succeed Dr. Charles B. Penrose, resigned. Dr. Clarke is a graduate of the University of Pennsylvania, in 1891, and for six years past has been connected with the Johns Hopkins Hospital.

---

ACCORDING to the Lancet, the increase of the population of England in the last ten years has been at the rate of 7.5 per cent., while the number of qualified practitioners has increased at just three times that rate. There is one practitioner for every 1,272 people, and the Lancet thinks that this state of affairs makes it impossible for all the medical men to earn a livelihood.

---

DURING the recent army maneuvers in England, quite a large portion of the men were disabled on account of some slight trouble with their feet. This has resulted in the establishment of classes among the non-commissioned officers for instruction in chiropody. The men are instructed in the prevention of corns, blisters, bunions, chilblains, ingrowing toe-nails, and anything else which would incapacitate them for much marching.

---

THE South Dakota Board of Health has refused to grant certificates to several applicants to practice osteopathy in South Dakota. The ground for rejection of these applicants was that the schools mentioned, one of which was at Kirksville, Mo., and the other at Minneapolis, Minn., are not, according to the published rules of the osteopathic authorities, schools of osteopathy in good repute. We wonder where such will be found.

---

THE following officers were elected for the year 1899-1900 at the recent meeting of the Mississippi Valley Medical Association held in Chicago: President, Dr. Harold N. Moyer of Chicago; Vice-Presidents, Dr. H. A. Cordier of Kansas City, and Dr. S. P. Collins of Hot Springs; Secretary, Dr. Henry E. Tuley of Louisville; Treasurer, Dr. Dudley S. Reynolds of Louisville. The next meeting will be held at Asheville, N. C.

A NEWSPAPER VIEW OF THE DUM-DUM BULLET.—The Dum-Dum bullet derives its name from Dum-Dum, India, where it was first made. Its top is of brass, and hollow. When it strikes its victim it becomes umbrella shaped, and tears its way through the flesh, making a dangerous wound. Blood-poisoning sets in within thirty minutes after the bullet strikes.

***NOTICE.—This JOURNAL and THE INTERNATIONAL JOURNAL OF SURGERY one year for $1.00 cash, to new subscribers. Out-of-town checks not accepted unless 10c. is added for cost of collection.***

AMONG the recent gems of newspaper medicine is an article reproduced in the *Sun* from the *Glasgow Mail*, in which "a well-known medical man" is said to have "announced," among other things, that the reason for the greater frequency of appendicular inflammation in the male sex was their habit of sitting with the legs crossed. It might be well to ascertain the relative frequency of the disease among tailors and among other men.

ABOUT one year ago the *Medical News* undertook the thorough investigation of the practices of Christian Science, placing the work in the hands of Dr. John B. Huber. The results of these investigations were published in the *Medical News*, beginning with the issue of January 21 and continuing in three succeeding numbers. We are pleased to note that Dr. Huber has extended his inquiries, and now publishes in the October number of *Appleton's Popular Science Monthly* a most convincing and readable summary of his work.

**NOTICE.—This JOURNAL and THE INTERNATIONAL JOURNAL OF SURGERY one year for $1.00 cash, to new subscribers. No out-of-town checks accepted unless 10c. is added for cost of cashing.**

Two drops of camphor on your tooth-brush will give your mouth the freshest, cleanest feeling imaginable, will make your gums rosy, and will absolutely prevent anything like cold sores or affections of the tongue. The gums, by the way, are barometers of our condition. If they are clear, bright and red, we are in good health, while, if our blood is thin and wanting in the mysterious red corpuscles that make us healthy, the gums will be pale pink, or if we are in a very bad way indeed, and much in need of a course of dialyzed iron, they will be almost white.

---

THE College of Physicians and Surgeons of Philadelphia, announces that the next award of the Alvarenga Prize, being the income for one year of the bequest of the late Senor Alvarenga, and amounting to about one hundred and eighty dollars, will be made on July 14, 1900, provided that an essay deemed by the Committee of Award to be worthy of the prize shall have been offered. The Alvarenga Prize for 1899 has been awarded to Dr. R. L. Randolph of Baltimore, Md., for his essay entitled, "The Regeneration of the Crystalline Lens—An Experimental Study."

---

### CHRISTIAN SCIENCE.

Oh, "God is so Good,"
If we sit down and brood
On the goodness and "Allness," within and without us,
We need have no fear,
Our crackers and beer
Will flow from the "Allness" and goodness about us.

Of course, "there's no evil"—
God's not so uncivil
To make us imperfect and send us to thunder.
"There nothing but love"—
In the heavens above,
The pockets of men, and the hearts that beat under.

"There can be no trouble,"
The body's a bubble—
It's all a "mistaken belief" and a dreaming.
God made us to fool us,
Till some one should school us
To see what we see to be only a seeming.

"We are nothing but spirit"—
We really don't hear it,
Or see it, or taste it, or smell it, or feel it,
"There is no sensation"—
Except the temptation
To think what we think when we think we can't heal it.

'Tis quite a mistaken
Idea we have taken,
That there's but one method of race propagation.
A child now to bother
About who's his father
Shows stubborn contempt for the new revelation.

"There's nothing but mind"—
Though created so blind
We're all of us nursing some little "delusion";
But friends by the score
(For a dollar or more)
Will kindly remove the distressing delusion.

With best of intention,
The Lord failed to mention—
While healing the halt and the deaf and the blind—
The trick of his healing
Was simply revealing
A "mortal deception" of "immortal mind."

And that these signs and wonders
Arose from the blunders
The Father had made in creating mankind;
And, until he was ready
To send Mrs. Eddy,
The world must remain to his purposes blind.

—*Medical Times.*

